# The mechanism and therapeutic prospect of HIF-1 α/BNIP3 pathway in regulating mitophagy in tubulointerstitial fibrosis

**DOI:** 10.1080/0886022X.2026.2667684

**Published:** 2026-05-05

**Authors:** Xinru Wang, Zhaoan Guo

**Affiliations:** ^a^ Clinical Medical College, Shandong University of Traditional Chinese Medicine, Jinan, China; ^b^ Affiliated Hospital of Shandong University of Traditional Chinese Medicine, Jinan, China

**Keywords:** Tubulointerstitial fibrosis, HIF-1α, BNIP3, mitophagy, pyroptosis

## Abstract

Tubulointerstitial fibrosis (TIF) is a key pathological hallmark and a major determinant of end-stage renal disease (ESRD). The mechanisms of TIF remain unclear, and there are currently no specific drugs to slow or reverse its progression. Notably, due to the kidney’s unique structure, the course of renal dysfunction is intimately connected with hypoxia. The signaling pathway formed by hypoxia-inducible factor 1α (HIF-1α) and its downstream target gene, B-cell lymphoma-2/adenovirus E1B 19-kDa interacting protein (BNIP3), exerts a pivotal effect during renal hypoxia. This pathway mediates mitophagy, inhibits apoptosis and inflammatory responses, maintains cellular energy balance, and thus profoundly influences the progression of TIF. This article focuses on the molecular mechanism by which the HIF-1α/BNIP3 pathway regulates mitophagy and affects TIF, and delves into its mechanisms in pyroptosis, oxidative stress, and ischemia-reperfusion injury, This work endeavors to establish a theoretical foundation and potential intervention targets for developing novel treatment strategies for TIF.

Tubulointerstitial fibrosis (TIF) is a major pathological manifestation of end-stage chronic kidney disease, and it is also a key standard to evaluate the trend of kidney disease. Hypoxia plays a central role in the occurrence and progression of TIF. Due to the high metabolic demand of renal tubules and the hypoxic microenvironment of medulla, the kidney is abnormally sensitive to ischemia and hypoxia. Hypoxia inducible factor 1 α (HIF-1 α), as an important factor regulating hypoxia, plays a key role in improving renal hypoxia by activating related genes and promoting angiogenesis [1]. Studies have confirmed that B-cell lymphoma-2/adenovirus E1B 19 kDa interacting protein (BNIP3) can be induced by HIF-1 α under hypoxia [2]. As an outer mitochondrial membrane protein, BNIP3 can directly bind to autophagy receptor binding microtubule associated protein light chain 3 (LC3), activate mitophagy, and promote damaged mitochondria to be phagocytosed and degraded [3], thereby reducing renal hypoxia reperfusion injury, decreasing cell death, and inhibiting TIF. In view of the extremely sensitive characteristics of TIF to ischemia and hypoxia, we believe that by regulating mitophagy mediated by HIF-1 α/bnip3 pathway, we can maintain the stability of the mitochondrial internal environment, and then inhibit the process of TIF. This paper systematically discusses the important role of HIF-1 α/BNIP3 pathway in inhibiting pyroptosis, oxidative stress and improving ischemia-reperfusion injury (IRI) by regulating mitophagy, in order to further explain the mechanism of TIF and seek possible therapeutic strategies.

## 1. Overview of the HIF-1α/BNIP3 pathway

### 1.1. Structure and function of HIF-1α

HIF is a heterodimeric protein composed of the α and β units. The α subunit is oxygen-dependent, while the β subunit is stably expressed intracellularly and is not regulated by oxygen levels. The HIF-1 family includes: HIF-1α, HIF-2α, and HIF-3α. Among them, HIF-1α is essential for both the cellular hypoxia response and the early adaptive response of tissue cells during hypoxia. Additionally, it is the member in clinical practice that has been researched the most [4]. HIF-1α’s N-terminal domain allows it to bind to DNA, and its C-terminal section contains an oxygen-dependent degradation activity that causes the ubiquitin-proteasome system to break down. Under normoxic situation, HIF-1α is transformed by prolyl hydroxylase (PHD), detected by the von Hippel-Lindau tumor suppressor protein (pVHL), and subsequently destined for proteasomal elimination *via* ubiquitination. However, in hypoxic conditions, PHD function is inhibited, preventing hydroxylation, which causes HIF-1α to stabilize and accumulate in the cytoplasm. The continuously accumulated HIF-1α combines with HIF-1β to create a dimer, which stimulate down genes targeted, such as BNIP3, erythropoietin, and glucose transporter-1. and allow cells to respond adaptively [5].

### 1.2. Mitophagy

Mitophagy, a targeted kind of autophagy, regulates mitochondrial amount and steady state by removing defective or dysfunctional mitochondria. Mitophagy primarily involves two classic pathways: the PTEN-induced kinase 1 (PINK1)/Parkin protein (Parkin) way and the receptor-dependent pathway. The PINK1/Parkin way is ubiquitin-mediated. When mitochondria function normally, translocases transport PINK1 to the inner mitochondrial membrane for cleavage and subsequent degradation, rendering it inactive. However, when mitochondria are damaged, translocase activity decreases, leading to PINK1 accumulation outside the membrane. This accumulation activates Parkin through phosphorylation, causing ubiquitination of extramembrane proteins and recruiting autophagy receptors that bind to LC3. This facilitates the absorption of mitochondria, fuses with lysosomes, and degrades the mitochondria [6]. If PINK1 and Parkin functions are lost, mitochondrial damage signals cannot be transmitted promptly, leading to the continuous accumulation of damaged mitochondria and ultimately cell death. The receptor-mediated way, also known as the non-ubiquitination-dependent way, mainly includes BNIP3, Nip3-like protein X, and the FUN14 domain containing 1. These three proteins can directly bind to LC3, promoting mitochondrial degradation. Although mitophagy activated by these three proteins is collectively referred to as the receptor dependent pathway, this pathway is mainly mediated by BNIP3, and BNIP3 is also the most relevant pathway to hypoxia. HIF-1α and hypoxia can both cause BNIP3. When renal tubular epithelial cells (RTECs) are damaged due to ischemia and hypoxia, BNIP3 is the first to respond and initially clears dysfunctional mitochondria. Therefore, studying the specific mechanisms of HIF-1α/BNIP3-mediated mitophagy is of particular importance. It is essential for maintaining homeostasis under hypoxic conditions and protecting cells from prolonged hypoxic injury.

### 1.3. HIF-1α/BNIP3 and mitophagy

HIF-1α, as a transcriptional regulator of BNIP3, is a key signaling molecule for activating mitophagy under hypoxic environments. On one hand, HIF-1α is the strongest stimulant for BNIP3 expression. Under hypoxia, HIF-1 α combines with HIF-1 β to form dimers and migrate to the nucleus, where it binds to target genes in BNIP3 and synthesizes BNIP3 protein. Then the transcribed BNIP3 protein can directly bind to LC3 and activate mitophagy. Screening of HIF-1α target genes showed that BNIP3 levels increased in tandem with hypoxia-induced increases in HIF-1α expression. On the other hand, LC3B-II, serves as a widely used marker for autophagy flux, is stably present in the autophagy structure throughout the entire autophagy process (including autophagosome formation and lysosomal degradation) [7]. Experiments have shown that LC3B is regulated by HIF-1α. Knockout of HIF-1α not only reduces BNIP3 levels, but also reduces LC3B levels accordingly [8]. Another study also found that hypoxia-inducible factor prolyl hydroxylase inhibitor (HIF-PHI) mediates the accumulation of BNIP3 and LC3B-II and enhances mitophagy levels by stabilizing HIF-1α [9]. In summary, the signaling pathway of hypoxia–HIF-1α–BNIP3–mitophagy has been well established.

However, due to the dual nature of HIF-1 α, the role of HIF-1 α/BNIP3 pathway mediated mitophagy in kidney is different. Under mild hypoxia, the HIF-1α/BNIP3 pathway is triggered, which encourages mitophagy. This not only inhibits apoptosis and improves organ tissue morphology, but also reduces the generation of reactive oxygen species (ROS) by clearing damaged mitochondria, thereby suppressing inflammatory responses and pyroptosis and maintaining energy metabolism balance. Under severe hypoxia, however, activation of this pathway and excessive mitophagy may conversely lead to an increase in mitochondrial-dependent apoptosis [10]. This article mainly discusses the positive role of the HIF-1α/BNIP3 pathway in inhibiting renal fibrosis, but also elaborates on the dual nature of HIF-1α in order to provide assistance for future drug development.

## 2. HIF-1α/BNIP3 and TIF

TIF is one of the signs of CKD to the end stage, and its characteristics are mainly related to the accumulation of extracellular matrix (ECM) and the activation of myofibroblasts. Specifically, TIF begins with damage to the RTECs. After RTECs is damaged, it undergoes epithelial-mesenchymal transition (EMT). This process causes RTECs to lose its epithelial cell characteristics and differentiate into a mesenchymal cell phenotype. At the same time, it releases profibrotic mediators, which promote the accumulation of ECM. Such repetition eventually led to the occurrence of TIF [11]. The key role of transforming growth factor-β1 (TGF-β1) in the formation of TIF is well known and widely studied by many researchers. However, because RTECs is rich in mitochondria, dependent on mitochondrial oxidative energy supply, and renal tubules have the characteristics of high metabolism and high oxygen consumption, the production and progression of TIF have also been proved to be related to HIF-1 α, aggregation of abnormal mitochondria, and lactic acid bacteria poisoning. When the kidneys are stimulated by hypoxia or other factors, the mitochondria in the cells are damaged. Subsequently, the damaged mitochondria release ROS, which activates ways such as nuclear factor kappa-B (NF-κB) and TGF-β/Smad, upregulates the expression of key pro-fibrotic factors such as connective tissue and vascular endothelial growth factor, thereby directly activating fibroblasts, promoting ECM deposition and TIF [12]. Additionally, stimulating the HIF-1α/BNIP3 promptly during early hypoxia can initiate mitophagy, block the release of ROS, and thus alleviate renal fibrosis. Therefore, timely activation of the HIF-1α/BNIP3 pathway in the early stages of hypoxia can block this pathway before damaged mitochondria release ROS, ultimately achieving the goal of protecting renal function and inhibiting TIF. Li et al. [1] demonstrated HIF-1α/BNIP3-dependent mitophagy stimulation exists in the UUO model, *via* immunoblotting analysis. Immunofluorescence results also proved the formation of phagosomes during hypoxia. In addition, the knockout of BNIP3 resulted in the damage to mitochondrial structure, matrix swelling, increased ROS, and aggravated fibrosis in mice, further confirming the importance of HIF-1α/BNIP3-caused mitophagy in maintaining mitochondrial function and inhibiting renal fibrosis. Furthermore, studies have shown that inhibition of HIF-1α/BNIP3-mitophagy can also activate the TGF-β1/p38 mitogen-activated protein kinase inflammatory pathway, exacerbating renal cell apoptosis and kidney damage. This further confirms that HIF-1 α/BNIP3 pathway is an important factor to inhibit TIF and protect kidney function [13]. Therefore, in-depth research into the mechanisms by which HIF-1α/BNIP3 activates mitophagy can provide new ideas and targets for a deeper understanding of the TIF mechanism, seeking possible treatment options, and ultimately delaying the progression of CKD.

### 2.1. HIF-1α/BNIP3 and pyroptosis

Pyroptosis is an inflammation related nontraditional form of cell death. Its molecular mechanism is different from traditional cell death. This process starts with the activation of nod like receptor protein 3 (NLRP3) inflammasome. Under the action of external stimuli, NLRP3 is activated, oligomerizing and recruiting apoptosis-associated speck-like protein containing a caspase recruitment domain (ASC), as well as cysteinyl aspartate specific proteinase-1 (caspase-1). ASC, NLRP3, and caspase-1 together constitute the NLRP3 inflammasome. Once the NLRP3 inflammasome is activated, caspase-1 can play multiple roles. On the one hand, it can produce inflammatory factors such as Interleukin-18 (IL-18), IL-1 β through its own cleavage. On the other hand, caspase-1 can selectively cleave its downstream protein gasdermin D (GSDMD), which results in GSDMD producing the pore’s N-terminal shape. And then the cell membrane’s integrity will be compromised by the exposed GSDMD-N-terminus, releasing IL-18, IL-1β, and other cellular contents of the inflammasome from the membrane pores and triggering a severe local inflammation [14]. In addition, local inflammatory responses can spread to neighboring cells and trigger pyroptosis in neighboring cells, exacerbating the body’s inflammatory effects. Studies have found that pyroptos is not only encourages the expulsion of inflammatory cytokines, but its activation directly contributes to the generation and development of TIF [15]. Additionally, Multiple studies have demonstrated a favorable correlation between the degree of renal fibrosis and higher caspase-1 and NLRP3 levels. For example, in mice with UUO and nephrectomy, researchers found that the level of NLRP3 inflammasomes increased significantly with the severity of obstruction [16], and administering NLRP3 inhibitors such as psoralen and quercetin to mice can effectively reduce the degree of TIF in mice [17]. These studies collectively confirm that NLRP3 inflammasome-mediated pyroptosis is one of the important mechanisms in the development of renal fibrosis. Therefore, targeting and inhibiting this pathway may provide a new therapeutic strategy for alleviating TIF in clinical practice.

Studies have confirmed that the expression of NLRP3 is mediated by HIF-1 α under hypoxic conditions [18]. Cosin-Roger et al. [19] also found the binding site of HIF-1 α at the −150 site of NLRP3 promoter region through chromatin immunoprecipitation.

**Figure 1.**
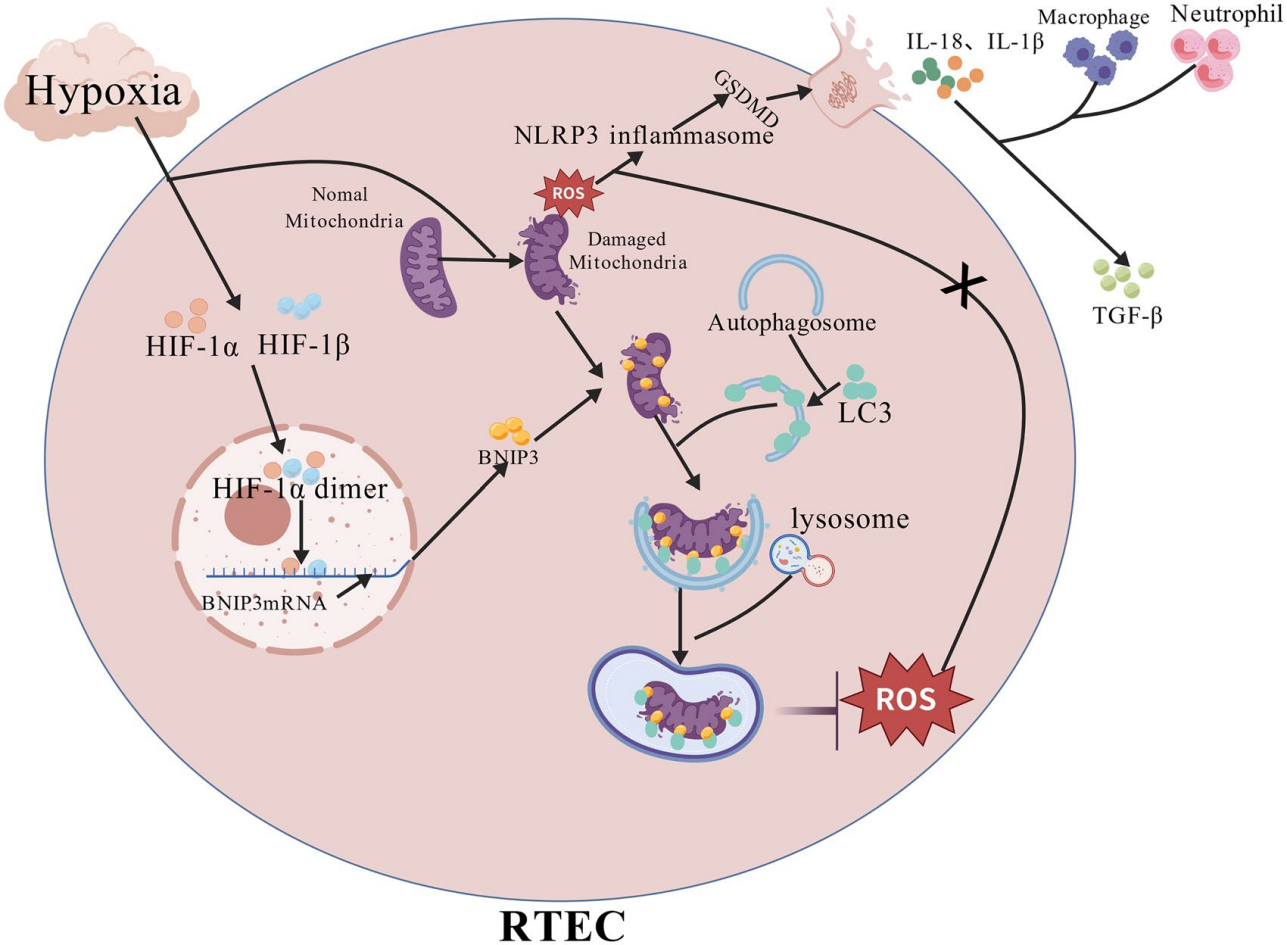
Mechanism by which HIF-1α/BNIP3 activates mitophagy and inhibits fibrosis.

In addition, the activation of NLRP3 inflammasome has also been confirmed to be related to the accumulation of damaged mitochondria in RTECs: ROS secreted by damaged mitochondria is important signals to trigger NLRP3 inflammasome. It is worth noting that HIF-1 α can also activate the expression of BNIP3 under hypoxia. Based on the above mechanism, we believe that HIF-1 α/BNIP3 can play a renal protective role by inducing mitophagy, clearing dysfunctional mitochondria, preventing the release of ROS, and then blocking the activation of NLRP3 inflammasome mediated pyroptosis, inhibiting TGF - β/Smad pathway [20]. The specific mechanism is shown in Figure 1. Li et al. [1]studied a UUO mouse model under low oxygen levels, not only proved the existence of the HIF-1α/BNIP3 pathway and the activation of mitophagy under low oxygen levels, but also confirmed the function of this pathway by knockdown of BNIP3. The results showed that after HIF-1 α/BNIP3 was inhibited, the level of mitophagy decreased, while the level of NLRP3 inflammasome increased. NLRP3 can activate GSDMD, rupture the cell membrane, induce the release of inflammasomes into the extracellular space, continuously activate interstitial inflammation, and release TGF - β 1 at the same time, ultimately promoting the process of fibrosis. After using mitochondrial antioxidants and NLRP3 inhibitors, not only could the decrease in the level of mitophagy caused by BNIP3 knockout be significantly improved, but the expression of NLRP3 inflammasomes could also be significantly downregulated, and the level of TIF could be improved. In contrast-induced acute kidney injury models, it was also discovered that the levels of proteins related to apoptosis and cytochrome C increased in cells after renal injury. Excessive ROS also led to a significant increase in the number of NLRP3 inflammasomes and caspase-1. Subsequently, ROS also activated the HIF-1α/BNIP3 pathway and mitophagy, enabling cells to make hypoxia-adaptive responses by maintaining mitochondrial quality, thereby limiting the diffusion of renal inflammation and mitigating renal injury and obstruction [21]. Therefore, augmenting the levels of HIF-1α or BNIP3 may enable mitophagy and concurrently inhibit the pyroptosis pathway, thereby improving the pathological course of TIF.

### 2.2. HIF-1α/BNIP3 and oxidative stress

The mechanisms by which oxidative stress leads to TIF are diverse. On the one hand, oxidative stress stimulates the expression of ROS, and excessive ROS expression damages podocytes and intracellular lipids and DNA, causing senescence and apoptosis in RTECs. More importantly, ROS can trigger a number of pro-fibrotic and pro-inflammatory ways, most notably the TGF-β1 signaling pathway. Continuous TGF-β1 stimulation causes renal cells to undergo EMT and encourages the buildup of extracellular matrix. In addition, increased ROS can also activate the NLRP3 inflammasome, promote pyroptosis, and release inflammatory factors such as IL-18, forming an inflammatory microenvironment. Together, these elements cause fibrous tissue to accumulate in the kidney [22,23]. On the other hand, the activation of oxidative stress will lead to the mutation of mitochondrial DNA, affect the synthesis of ATP, and disrupt the homeostasis of Ca^2+^. As an important messenger, Ca^2+^ can regulate the actin dynamics of renal downstream cells such as RTECs and podocyte, Disruption of Ca^2+^ homeostasis leads to podocyte shedding, accelerating glomerular sclerosis. Abnormal Ca^2+^ can also activate endoplasmic reticulum stress, induce the secretion of profibrotic mediators such as TGF-β1, stimulate myofibroblast phenotypic transformation, and ultimately trigger TIF [24].

One of the fundamental characteristics of oxidative stress is an imbalance of ROS, and mitochondria, the primary structure that generates ROS, are crucial in controlling cellular oxidative stress. Under hypoxic conditions, impaired mitochondrial function leads to increased peroxide production, which in turn triggers oxidative stress, exacerbates inflammatory responses and promotes cell death. At the same time, hypoxia can also upregulate the level of BNIP3 by accumulating HIF-1α, inducing the initiation of mitophagy, thereby effectively removes dysfunctional mitochondria, reducing the accumulation of ROS, and alleviating inflammatory responses and oxidative stress damage [1]. Jia et al. [25] found that UUO rats showed increased ROS levels, and loss of enzymes that are antioxidants, mitochondrial damage, and unbalanced mitochondrial dynamics. Treatment with Tongluo Yishen Decoction alleviated the quantity of ROS and the severity of TIF, as well as UUO-induced oxidative stress. Further research revealed that the renal protective effect of Tongluo Yishen Decoction is related to its significant activation of mitophagy and clearance of dysfunctional mitochondria. In addition, knockout of HIF-1α has been shown to significantly increase ROS levels and exacerbate cellular oxidative stress damage, while increased expression of BNIP3 can reverse this phenomenon, reduce autophagy inhibition caused by HIF-1α knockout, and inhibit ROS expression by selectively expelling dysfunctional mitochondria, consequently lowering the degree of renal function decline, renal fibrosis, and oxidative stress. In conclusion, we believe that activation of mitophagy through the HIF-1α/BNIP3 pathway plays a crucial controlling role in alleviating oxidative stress damage and inhibiting TIF.

### 2.3. HIF-1α/BNIP3 and apoptosis

Apoptosis is essential for preserving cellular homeostasis and proper function by eliminating senescent and harmful cells. Under physiological conditions, mitophagy and apoptosis influence each other, maintaining a dynamic balance. However, when mitophagy is inhibited, apoptosis is promoted, leading to disease. Abnormal mitophagy results in swollen and fragmented mitochondria, leading to reduced ATP synthesis and a rise in ROS. It also reduces the level of anti apoptotic protein Bcl-2 in the cytoplasm, increases the expression of Pro apoptotic factor Bax, and translocates Bax to the mitochondrial membrane, causing the increase of mitochondrial outer membrane permeability and promoting the release of cytochrome C in mitochondria into the cytoplasm. Subsequently, the released cytochrome C, caspase-9 precursor and apoptotic protease activator-1 form apoptotic bodies. The apoptotic bodies cleave and activate caspase-3 precursor to produce active caspase-3. Caspase-3 activates DNase, breaks DNA, and finally causes apoptosis [26]. Apoptosis plays an important role in promoting the development of TIF. Apoptotic cells release cytokines such as tumor necrosis factor-α and TGF-β1, leading to inflammatory response and TIF [27]. The study also found that the use of anti apoptotic protein AKT1 could not only significantly reduce the levels of active caspase-3 and Bax genes in unilateral IRI mice, but also significantly reduce the degree of EMT and TIF in mouse renal tubules [28]. Therefore, inhibiting the occurrence of apoptosis can reduce the inflammatory signals produced by apoptotic cells, reduce the expression of pro apoptotic factors and pro fibrotic factors in renal cells, and then inhibit the occurrence of TIF.

Mitophagy mediated by HIF-1α/BNIP3 is essential for preventing apoptosis. The activation of mitophagy mediated by this pathway can clear damaged mitochondria and ROS, block the apoptotic pathway, protect RTECs, and prevent the occurrence of fibrosis before the release of cytochrome C. Wang et al. [9]found that HIF-PHI can upregulate BNIP3-mediated mitophagy by stabilizing HIF-1α and reduce RTECs inflammation and apoptosis. To learn more about whether this effect is achieved by upregulating BNIP3-mediated mitophagy, the experimental group first used mdivi-1 to inhibit the occurrence of mitophagy in mice, and found that the anti apoptotic effect mediated by hif-phi was reversed by the use of mitophagy inhibitors. Subsequently, in order to eliminate the interference of Parkin-dependent pathways and other factors on the experiment, the experimental group knocked out BNIP3 in mice. It was also found that BNIP3 knockout not only reversed the accumulation of LC3B-II induced by HIF-PHI, but also eliminated the effect of HIF-PHI in inhibiting inflammation and apoptosis. Therefore, this experiment demonstrates that HIF stability can inhibit RTECs apoptosis and protect kidney function by activating BNIP3 and mediating mitophagy. In addition, immunoblot and TUNEL staining analysis showed that the knockdown of BNIP3 and HIF-1 α in mouse renal cortex would lead to a significant increase in the number of apoptotic cells, and rosalastat treatment was proved to reduce iohexol induced apoptosis and kidney injury by upregulating HIF-1 α/BNIP3 mediated mitophagy [8]. Another study by Li et al. [29] also found that the use of cisplatin can reduce the viability of HK-2 cells (human RTECs), cause apoptosis, damage and fibrosis. However, the treatment of Panax notoginseng saponin can increase HIF-1 α, BNIP3 and activate mitophagy, which not only increases the mitochondrial membrane potential and ATP levels reduced by cisplatin, significantly reduces the levels of apoptosis related proteins and mitochondrial apoptosis, but also improves the viability of HK-2 cells and reduces cisplatin induced acute kidney injury and TIF levels.

## 3. HIF-1α/BNIP3 and IRI

As a highly perfused organ, the kidney is highly sensitive to ischemia. IRI often occurs after kidney transplantation or heart surgery. It is an important inducement that leads to a sharp decline in renal function and causes acute kidney injury and TIF. Renal IRI mainly includes ischemia injury and reperfusion injury, which not only refers to the damage of renal structure and function caused by sudden interruption of blood flow, but also includes further damage to the kidney after the resumption of blood flow interruption [30]. Acute, transient hypoxia stimulates the production of HIF-1α, thereby activates downstream target genes of HIF-1α and exerting a nephroprotective effect. However, persistent ischemia and hypoxia can cause excessive activation of immune cells, leading to the massive release of pro-fibrotic factors such as TGF-β1 and α-smooth muscle actin, as well as excessive fibrosis, which is a key inducing factor for TIF [31]. If renal IRI can not be effectively controlled, the disease will rapidly progress to the end stage, and even cause the death of patients. Therefore, understanding the mechanism of renal IRI and seeking appropriate treatment measures can not only provide a new treatment for TIF, but also reduce the incidence of CKD and the mortality rate.

Mitophagy plays a crucial role in alleviating IRI and preventing TIF by specifically recognizing and clearing damaged mitochondria. The evidence that HIF-1 α/BNIP3 pathway activates autophagy to alleviate kidney injury during renal IRI is multifaceted. On the one hand, in the acute kidney injury model, it was found that the loss of mitophagy in proximal renal tubules would lead to mitochondrial swelling and damage in RTECs, as well as elevated production of pro-apoptotic proteins in mitochondria. HIF-1α can induce mitophagy by activating BNIP3, thereby reducing apoptosis in RTECs and protecting ischemic kidneys [32]. On the other hand, in a bilateral renal artery ligation model of diabetic nephropathy (DN), it was discovered that the use of HIF-1α inhibitors suppressed the HIF-1α/BNIP3 pathway and mitophagy, and ROS generation also increased. However, when the Brain and Muscle ARNT-Like 1 gene (Bmal1), a core component of the mammalian circadian clock, was administered to mice, the HIF-1α/BNIP3 axis and mitophagy were activated, and cell damage and apoptosis were significantly reduced [33]. Similarly, renal injury caused by IRI was also alleviated after using HIF-PHI or directly injecting recombinant HIF-1 α protein [34]. *In vitro* and *in vivo* studies further indicate that HIF-1α can not only exert a nephroprotective effect through the HIF-1α/BNIP3 pathway, but it can also exert a protective effect in the kidneys of IRI by inducing downstream genes such as vascular endothelial growth factor, heme oxygenase 1, and erythropoietin. Notably, HIF-1α/BNIP3-dependent mitophagy has been shown to maintain the function of the mitochondrial, thus lowering ROS production. When BNIP3 inhibitors are used, renal IRI is also aggravated [35].

In summary, stimulation of the HIF-1α/BNIP3 axis and upregulation of mitophagy activity can provide new targets and ideas for the prevention and treatment of renal IRI, thereby preventing the occurrence of TIF and the progression of CKD.

## 4. The dual nature of HIF-1α

Although numerous researches have demonstrated that the HIF-1α/BNIP3 pathway can inhibit the progression of TIF by activating mitophagy, the effects of HIF-1α on TIF and chronic kidney disease remain controversial. HIF-1α itself exhibits significant functional duality, which primarily depends on the degree of kidney damage, the time and extent of HIF-1 α activation, cell type, and its downstream signaling pathways.

### 4.1. The degree of kidney damage and the time and extent of HIF-1 α activation

The effect of HIF-1 α on TIF is closely related to the severity of kidney damage and the degree of HIF-1 α activation. On the one hand, early, acute and moderate activation of HIF-1 α can protect renal function. At this time, the transient ischemic and hypoxic period will promote the stable expression of HIF-1 α. HIF-1 α will activate mitophagy by upregulating BNIP3, timely remove dysfunctional mitochondria, reduce the production of ROS from the source, and then inhibit the activation of inflammasomes such as TGF-β, NF-κB, and NLRP3, playing a renoprotective role. However, late-stage, chronic, and persistent hypoxia stimulates the overexpression of HIF-1α. While HIF-1α also upregulates BNIP3, its overexpression stimulates the increase of insulin-like growth factor binding protein 3, thereby inhibiting the phosphatidylinositol 3-kinase/protein kinase B pathway and increasing renal cell apoptosis. In addition, under continuous hypoxic stimulation, the sustained activation of HIF-1 α also drives abnormal metabolic reprogramming, which will not only promote the occurrence of EMT and the metastasis of epithelial cells, but also increase the infiltration of inflammasomes such as NLRP3, the accumulation of TGF-β, the expression of fibroblasts in the kidney, and ultimately promote the development of TIF [36]. For example, in the study on the intervention of HIF-1 α in UUO mice, it was found that severe obstructive hypoxia would upregulate the expression of HIF-1 α in mice, induce abnormal mitochondrial quality control, and trigger metabolic reprogramming, specifically manifested as the increase of intracellular glycolytic metabolites and the decrease of ATP. These metabolic disorders would eventually trigger downstream inflammation related mechanisms, leading to the occurrence of TIF [37]. In the IRI model, moderate activation of HIF-1α can reduce inflammation and exert a renal protective effect [38]. On the other hand, the effect of HIF-1 α on TIF is also closely related to the degree of kidney damage. Severe and persistent kidney injury, such as persistent ischemia and hypoxia, can inhibit the activity of nuclear factor erythroid derived-2-related factor 2 [39], and also lead to irreversible damage of renal tubules and the release of exosomes. These exosomes will stabilize HIF-1 α in cells, promote glycolysis and the expression of TGF-β, IL-1 β, etc., and aggravate inflammation and fibrosis [40]. Finally, the imbalance between the degree of kidney damage and the expression of HIF-1 α is also an important factor leading to TIF injury. For example, when hypoxia and other stimuli lead to a large area of oxidative stress and apoptosis in the kidney, the renal tissue damage is more serious. At this time, the insufficient expression of HIF-1 α is the key pathogenic factor: due to the lack of HIF-1 α, the inflammation and oxidative stress in RTECs cannot be effectively suppressed, resulting in the persistent and expanding damage. In addition, HIF-1 α, as a key leader in the repair of damage under hypoxia, its lack also makes the cell repair mechanism unable to function normally, and eventually aggravates kidney damage [41].

### 4.2. Other factors

The level of HIF-1α deacetylation, cell type, and triggered downstream molecules are also important reasons for the duality of HIF-1α. Sirtuin 1 (SIRT1), as a NAD^+^ driven deacetylase, can inactivate target proteins by deacetylation. It has been reported that activation of the SIRT/HIF-1α signaling pathway can inhibit and alleviate kidney tissue damage [42]. SIRT can remove the acetyl group on HIF-1α by deacetylation, thereby altering the transcriptional activity of HIF-1α and exerting an inhibitory effect on fibrosis. Conversely, in the late stage of the disease or when SIRT1 activity is weakened, it is unable to deacetylate HIF-1α. If HIF-1α accumulates excessively at this time, it will lead to the initiation of EMT, glycolysis, etc, thus exhibiting a pro-fibrotic effect [43]. Studies have also shown that by using SIRT agonists to intervene in DN rats, SIRT involvement can downregulate HIF-1α, reduce the release of inflammatory factors, and repair podocyte damage [36]. Secondly, HIF-1α is distributed differently in various kidney cells, In addition, the sensitivity of varies renal cells to hypoxia is different, so the activity of HIF-1 α after activation is different, which ultimately leads to its varies role. For example, under normal circumstances, proximal RTECs are highly sensitive to hypoxia, but HIF-1α is less allocation in proximal RTECs. When hypoxia or other stimuli occur, HIF-1α in them will undergo drastic dynamic changes. If HIF-1α rises sharply, it is more likely to exert a pro-fibrotic effect, and conversely, it will exert an anti-fibrotic effect [44]. In addition, overexpression of HIF-1α can increase the level of downstream NADPH oxidase 4, which in turn causes an rise in ROS and oxidative stress. The rise in ROS is also an important initiating factor for the activation of HIF-1α. Thus, such repetition leads to continuous amplification of fibrosis signal [45].

In summary, HIF-1α has a dual nature in renal fibrosis; whether its ultimate effect is pro-fibrotic or anti-fibrotic depends on its activation level, duration, and the synergistic regulation of multiple other factors. This complexity underscores the profound research value of HIF-1α-related pathways and indicates that the key to future research lies in the precise regulation of its activity. Despite the complexity of the HIF-1α pathway mechanism, therapeutic exploration targeting this pathway has shown clinical potential. For example, in the IRI model, it was found that the application of HIF-PHI such as roxadustat can activate the HIF1α/BNIP3 pathway, enhance mitophagy, thereby alleviating drug-induced kidney injury, suppressing renal cell apoptosis, and promoting renal function and the degree of renal fibrosis [8]. Therefore, in-depth research and precise regulation of HIF1α/BNIP3-mitophagy pathway still have important clinical significance and prospects.

## 5. Summary and outlook

This paper systematically searched PubMed, CNKI and other databases to sort out and summarize the related research on HIF-1 α/BNIP3 pathway, mitophagy and TIF in recent years. At present, there is controversy about the mechanism of HIF1 α in TIF: acute and moderate activation can play a protective role, while chronic excessive activation can promote TIF through EMT and other pathways. Nevertheless, many studies have consistently confirmed that HIF1 α/BNIP3 can activate mitophagy, reduce ROS generation, inhibit pyroptosis, oxidative stress, etc., and then exert inhibitory effects on TIF. It is worth noting that BNIP3 mediated mitophagy responds more rapidly to the stimulation of hypoxia, and BNIP3 is regulated by HIF1 α. Therefore, hypoxia-HIF-1 α - BNIP3 mitophagy, a complete signaling axis, can provide a unique molecular basis for the development of drugs. At present, loxastat and other HIF-PHI have been widely used in the clinical treatment of renal anemia, but its curative effect on TIF is not clear. Therefore, in future studies, we can further develop drugs that target HIF1 α/BNIP3 mitophagy TIF, and focus on the different effects of HIF1 α on the kidney under different HIF1 α thresholds. This can not only help us alleviate TIF, but also stop the process of CKD and optimize the prognosis of patients.

## Data Availability

Data sharing is not applicable to this article as no new data were created or analyzed in this study.
